# Case Report: Hermansky-Pudlak syndrome type 1 in the Russian Federation presenting with genotype-phenotype discordance

**DOI:** 10.3389/fped.2026.1946494

**Published:** 2026-10-28

**Authors:** L. G. Khachatryan, N. V. Chincharauli, G. S. Golosnaya, K. A. Kazakova, N. S. Trifonova, V. A. Kaptilnyy, M. V. Poznyak, R. A. Zinchenko, A. S. Kulagin, S. A. Ionova, E. V. Zhukova, E. V. Minashkina, O. S. Lukyanchuk, R. A. Chilova

**Affiliations:** 1 Sechenov University, Moscow, Russia; 2 Research Centre for Medical Genetics, Moscow, Russia

**Keywords:** case report, Hermansky-Pudlak syndrome, HPS1 gene, iris transillumination, oculocutaneous albinism, platelet storage pool deficiency

## Abstract

**Background:**

Hermansky-Pudlak syndrome (HPS) is a rare autosomal recessive disorder caused by defects in the biogenesis of lysosome-related organelles (LROs) across multiple tissues. This condition typically presents with a triad of oculocutaneous albinism, platelet dysfunction, and visceral complications. Hermansky-Pudlak syndrome type 1 (HPS-1), caused by pathogenic variants in the HPS1 gene, carries a high risk for progressive pulmonary fibrosis.

**Case presentation:**

This case report describes a genetically confirmed case of HPS-1 in a 10-year-old girl with an unusual clinical phenotype, particularly regarding ophthalmological and gynecological findings. She presented with oculocutaneous albinism, bleeding diathesis despite normal platelet counts, foveal hypoplasia, and an atypical absence of iris transillumination (score 0/5 on a standardized scale). Next-generation sequencing (NGS) identified compound heterozygous pathogenic variants in the HPS1 gene (NM_000195.5:c.397G > T and c.972dup). Pelvic ultrasound revealed marked uterine hypoplasia.

**Conclusion:**

This case describes a rare phenotype associated with these HPS1 variants, illustrating the clinical variability of the syndrome. Our findings support the importance of NGS-based diagnostics and the need for multidisciplinary long-term monitoring, including ophthalmological, pulmonary, gastrointestinal, and hematological surveillance. Reporting atypical presentations is important for improving our understanding of the clinical spectrum of rare disorders such as HPS, with national registries serving as a valuable tool to collect such rare phenotypic data.

## 1 Introduction

Hermansky-Pudlak syndrome (HPS) is a rare autosomal recessive disorder caused by defects in the biogenesis of lysosome-related organelles (LROs) (1). This condition is characterized by a classic triad of oculocutaneous albinism (OCA), platelet dysfunction marked by bleeding diathesis, and visceral manifestations such as pulmonary fibrosis, granulomatous colitis, and, in certain subtypes, neutropenia (2). Currently, 11 HPS subtypes have been identified. Each subtype is associated with pathogenic variants in genes encoding subunits of the adaptor protein-3 (AP-3) complex and LRO biogenesis complexes (BLOC-1, BLOC-2, BLOC-3) (3). HPS type 1 (HPS-1), caused by biallelic variants in the *HPS1* gene (locus 10q24.2), is the most common form. Recent epidemiological data indicate a global prevalence of 1–9 cases per 1,000,000 population, though in Puerto Rico, the prevalence reaches approximately 1:1,800 due to a well-documented founder effect (4).

This case report describes a molecularly verified case of HPS-1 notable for an atypical ophthalmological phenotype (absence of iris transillumination) and comorbid uterine hypoplasia. This observation demonstrates an unusual genotype-phenotype discordance in comparison to previously reported patients with identical *HPS1* variants (5), expanding the recognized phenotypic spectrum of the syndrome. This report highlights the need for a multidisciplinary diagnostic approach, underscores that absent iris transillumination does not exclude HPS, and aligns long-term monitoring algorithms with current clinical guidelines.

## 2 Case description

### 2.1 Patient information and medical history

Patient D., aged 10 years and 1 month at the time of evaluation, was born from her mother's sixth pregnancy, which was complicated by acute pyelonephritis during the second trimester requiring antibiotic treatment. She was delivered via elective cesarean section at 39 weeks of gestation with a birth weight of 3,200 g, a length of 48 cm, and Apgar scores of 8 and 9. Routine neonatal and audiological screenings revealed no abnormalities, though divergent strabismus was noted from birth. During her first two months, she experienced feeding difficulties with frequent regurgitation, which resolved after transitioning from breast milk to an anti-reflux formula.

At 1 month of age, the patient developed horizontal nystagmus, prompting an ophthalmological evaluation. By 3 months, she was diagnosed with OCA; photochromic glasses and regular clinical monitoring were recommended. Starting at 1 year of age, she developed signs of a bleeding diathesis, including frequent ecchymoses with delayed resolution (up to 3 weeks), periodic epistaxis, and prolonged bleeding following dental procedures. The bleeding phenotype was retrospectively quantified using the International Society on Thrombosis and Haemostasis Bleeding Assessment Tool (ISTH-BAT), yielding a score of 6 (indicating a clinically significant bleeding diathesis) (6). At age 2, she experienced an episode of acute pyelonephritis. From age 3, she had recurrent upper respiratory tract infections (up to 7 episodes annually), frequently by fever, vomiting, and diarrhea.

A hematology consultation at age 7 revealed no significant abnormalities on routine laboratory tests. Given the overall clinical presentation, HPS was suspected at 7 years and 10 months of age, and genetic testing was recommended. At age 10, the patient presented with a 3-week history of lower abdominal pain. Abdominal and pelvic ultrasound demonstrated a gallbladder fold, bilateral renal pelvic dilation, and an infantile uterus suggestive of uterine hypoplasia. A timeline of the patient's clinical milestones from birth to current evaluation is summarized in Figure 1.

**Figure 1:**
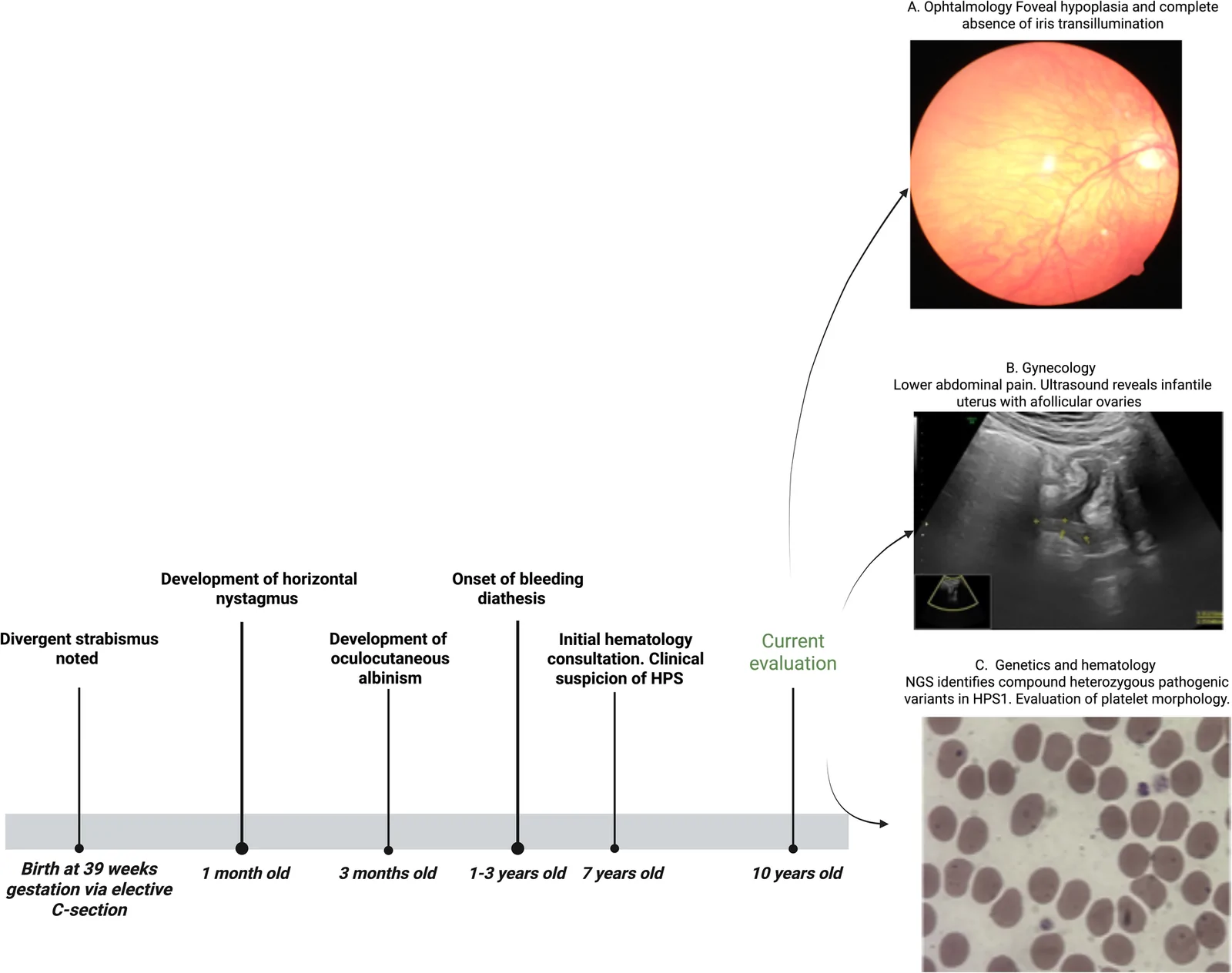
Clinical timeline of the patient. The timeline highlights key clinical milestones from birth to 10 years of age, illustrating the delayed diagnosis typical for HPS-1. The current evaluation (age 10) integrates molecular genetic confirmation with key phenotypic findings: foveal hypoplasia on fundus examination and morphological evaluation of platelets on a peripheral blood smear.

### 2.2 Physical examination

On physical examination, the patient's growth parameters were proportionate and ageappropriate (height, 139 cm; weight, 37.2 kg; body mass index, 19.2 kg/m^2^). She was hemodynamically stable and breathing comfortably on room air, with a blood pressure of 105/75 mmHg, a heart rate of 96 beats per minute, a respiratory rate of 20 breaths per minute, and an oxygen saturation of 99%.

Physical findings included dry, depigmented, and photosensitive skin, silvery-yellow hair and eyebrows, and signs of keratosis pilaris. She also had multiple resolving ecchymoses on her lower extremities and buttocks, as well as a 1.5 × 1.5 cm hemangioma and nevi scattered on the anterior chest wall.

The oropharynx was clear. Cardiopulmonary assessment demonstrated a regular heart rhythm and clear breath sounds bilaterally. Her abdomen was soft and non-tender without hepatosplenomegaly, and she maintained normal bowel habits.

### 2.3 Ophthalmological status

The patient had an uncorrected visual acuity of 0.1 OD and 0.15 OS, alongside a horizontal finebeating nystagmus. Slit-lamp biomicroscopy showed a complete absence of iris transillumination (grade 0 on a standardized 5-point scale). Fundus examination (Figure 1, insert B) demonstrated an albinotic appearance with prominent peripheral choroidal vessels, and optical coherence tomography (OCT) confirmed foveal hypoplasia.

### 2.4 Genitourinary examination

The patient had normal external female genitalia. Her Tanner stage was I (B1, P1), consistent with a prepubertal, premenarchal state.

Pelvic ultrasound revealed a moderately echogenic, cylindrical uterus measuring 21.3 × 4.8 × 4.5 mm without a distinct endometrial stripe (Figure 2A). Given the normal reference length of 28.0 to 43.0 mm for a 10-year-old, these dimensions are comparable to those of a 1- to 3-year-old child. Both ovaries were afollicular, with volumes up to 1.0 cm^3^ (Figures 2B,C). These volumes, below the age-appropriate norm of 1.0–2.5 cm^3^, suggest constitutional delay of puberty. The vagina was not visualized, an expected prepubertal finding.

**Figure 2:**
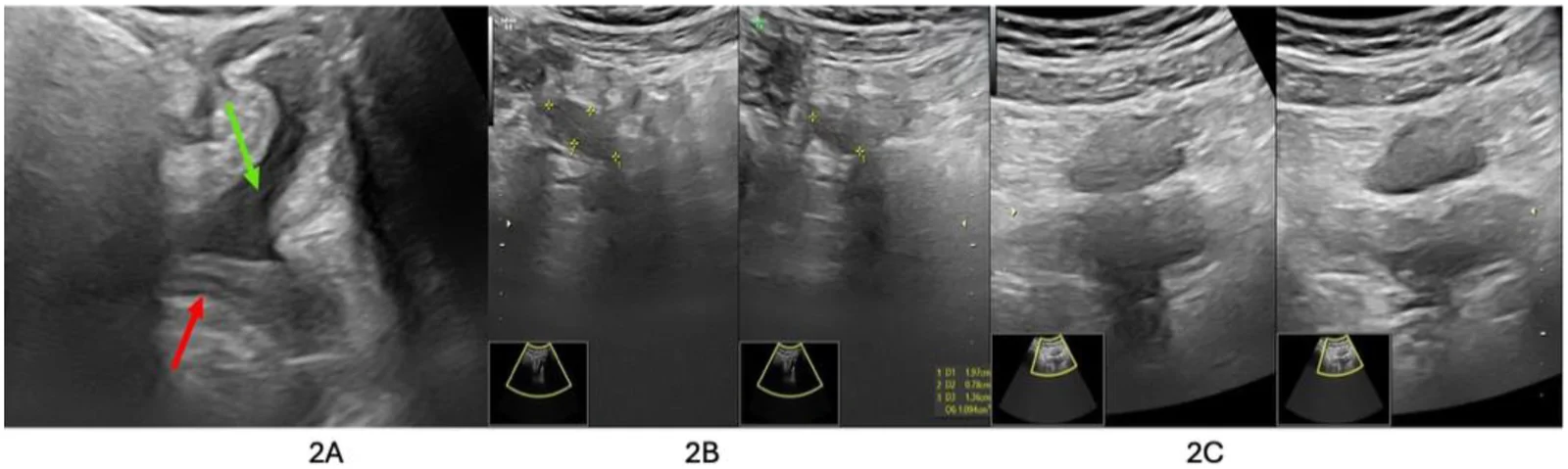
Transabdominal pelvic ultrasound findings. **(A)** Sagittal view highlighting the urinary bladder (green arrow) and the hypoplastic, cylindrical uterus (red arrow) measuring 21.3 × 4.8 × 4.5 mm, consistent with a prepubertal 1- to 3-year-old developmental age. **(B)** Right ovary, afollicular (volume 1.09 cm³). **(C)** Left ovary, afollicular, with volume and morphology consistent with a 5- to 6-year-old developmental age.

Additionally, renal ultrasound noted bilateral pyelectasis (up to 8 mm) without ureteral dilation-a likely transient condition requiring serial monitoring.

## 3 Diagnostic assessment

### 3.1 Laboratory findings

Complete blood count (CBC) analysis on the Sysmex XS-1000i system (Table 1) revealed normal red blood cell (RBC) and hemoglobin (HGB) levels, with a platelet (PLT) count of 216 × 10⁹/L (reference range, 184–512 × 10⁹/L). Automated counts were validated by peripheral smear analysis, accounting for citrate dilution. Morphological evaluation of the platelet profile indicated an elevated mean platelet volume (MPV) of 11.6 fL (reference range, 6.5–10 fL) and a normal plateletcrit (PCT) of 0.25%.

**Table 1:** Complete blood count, coagulation profile, and thromboelastography parameters.

Parameter	Result	Reference range*	Units	Comment
Complete blood count (Sysmex XS-1000i)				
White blood cells (WBC)	5.06	5.0–13.5	×10⁹/L	Normal
Red blood cells (RBC)	4.74	3.80–5.80	×10^12^/L	Normal
Hemoglobin (HGB)	137		g/L	Normal
Hematocrit (HCT)	37	33–45	%	Normal
Mean corpuscular volume (MCV)	78.1	69–93	fL	Normal
Mean corpuscular hemoglobin (MCH)	28.9	26–35	pg	Normal
Mean corpuscular hemoglobin concentration (MCHC)	37.0	32.0–36.0	g/dL	Mildly elevated
Red cell distribution width (RDW-CV)	33.1	—	%	—
Platelets (PLT)	216	184–512	×10⁹/L	Normal
Plateletcrit (PCT)	0.25	0.19–0.36	%	Normal
Mean platelet volume (MPV)	11.6	6.5–10.0	fL	Mildly elevated
Platelet distribution width (PDW)	16.0	9.0–17.0	%	Normal
Platelet large cell ratio (P-LCR)	38.4	10.7–45.0	×10⁹/L	Normal
Additional platelet verification				
Platelet count (EDTA, peripheral smear)	210	184–512	×10⁹/L	Automated count confirmed
Platelet count (Citrate, peripheral smear)	205	184–512	×10⁹/L	Adjusted forcitrate dilution
WBC differential (relative)				
Neutrophils (NE%)	43.1	45.0–72.0	%	Mildly decreased
Lymphocytes (LY%)	40.3	30.0–46.0	%	Normal
Monocytes (MO%)	15.4	3.0–12.0	%	Mildly elevated
Eosinophils (EO%)	0.8	1.0–5.0	%	Mildly decreased
Basophils (BA%)	0.4	0.0–1.0	%	Normal
WBC differential (Absolute)				
Neutrophils (NE#)	2.18	1.50–8.00	×10⁹/L	Normal
Lymphocytes (LY#)	2.04	1.20–5.20	×10⁹/L	Normal
Monocytes (MO#)	0.78	0.20–1.00	×10⁹/L	Normal
Eosinophils (EO#)	0.04	0.00–0.08	×10⁹/L	Normal
Basophils (BA#)	0.02	0.02–0.50	×10⁹/L	Normal
Erythrocyte sedimentation rate (ESR, Panchenkov method)	2	2–20	mm/h	Normal
Manual WBC differential (%)				
Band neutrophils	2	1–3	%	Normal
Segmented neutrophils	35	43–59	%	Mildly decreased
Lymphocytes	45	30–46	%	Normal
Monocytes	17	2–12	%	Mildly elevated
Eosinophils	1	0–5	%	Normal
Coagulation profile				
Prothrombinindex (PI)	87	80–120	%	Normal
Fibrinogen	2.38	1.8–4.0	g/L	Normal
Tromboelastography (TEG-5000)				
Reaction tine (R)	6.2	5–10	min	Normal
Clot formation time (K)	1.7	1–3	min	Normal
Maximum amplitude (MA)	61.9	50–70	mm	Normal
Lysisat 30 min (LY30)	0.2	≤ 0.8	%	Normal, low fibrinolysis

* Reference ranges are taken from standard clinical laboratory guidelines (Menshikov, 2008; Lugovskaya et al., 2014).

Coagulation studies and thromboelastography (TEG 5000) demonstrated parameters within normal limits (Figure 3), suggesting intact secondary hemostasis: reaction time (R) 6.2 min (reference range, 5–10 min), clot formation time (K) 1.7 min (reference range, 1–3 min), maximum amplitude (MA) 61.9 mm (reference range, 50–70 mm), and lysis at 30 min (LY30) 0.2% (reference range, ≤0.8%). This profile is consistent with the HPS-1 clinical profile characterized by qualitative platelet dysfunction (*δ*-granule deficiency) rather than coagulation factor defects.

**Figure 3:**
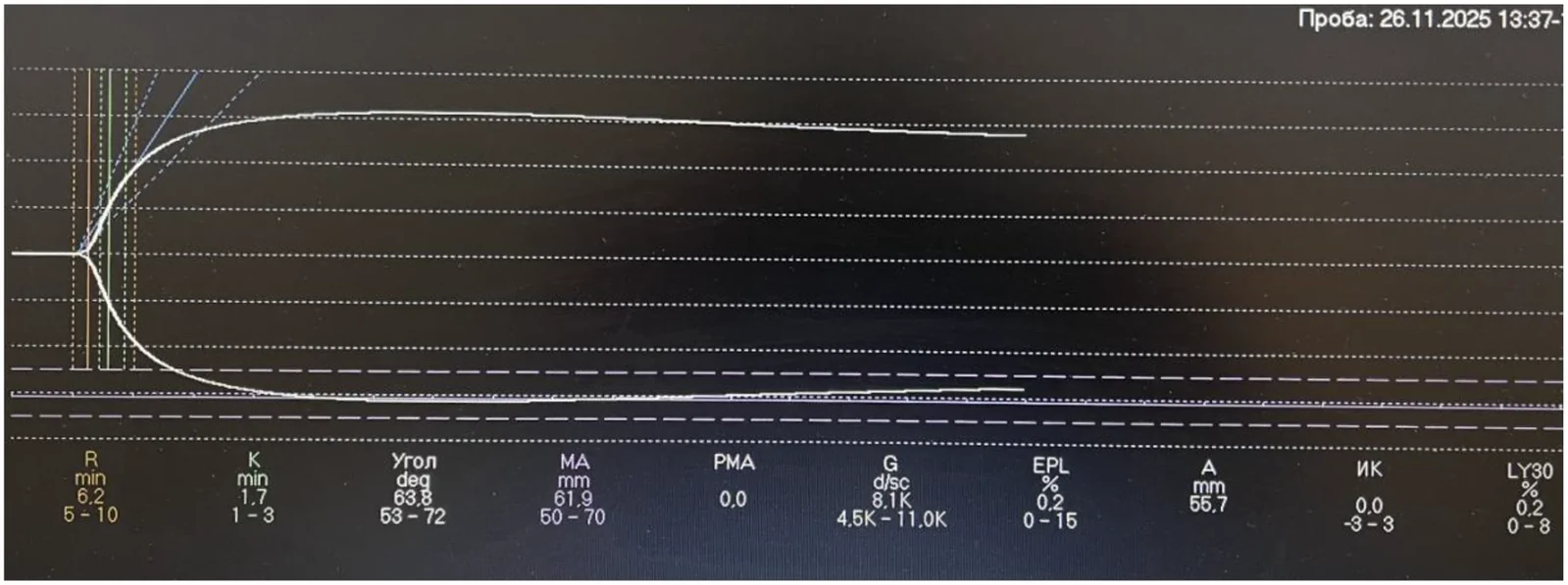
Thromboelastography (TEG 5000) tracing of the patient. The tracing confirms normal secondary hemostasis parameters (R = 6.2 min, K = 1.7 min, MA = 61.9 mm, LY30 = 0.2%), which is consistent with the HPS-1 clinical profile characterized by qualitative platelet dysfunction rather than coagulation factor defects.

#### 3.1.1 Clinical interpretation of notable deviations

- Mildly elevated MCHC (37.0 g/dL) and relative monocytosis (15.4%) likely reflect postinfectious changes or age-appropriate variation.
- All coagulation and TEG parameters are within normal limits. This is characteristic of HPS, where the defect strictly impairs qualitative platelet function (*δ*-granule deficiency) rather than platelet count or coagulation pathways (7).

Methodological limitation: Evaluating dense granules via light microscopy of a peripheral blood smear is not a validated method for diagnosing *δ*-granule deficiency. Verification of platelet dysfunction in HPS requires platelet aggregometry, flow cytometry for platelet activation markers, or electron microscopy (8, 9).

### 3.2 Molecular genetic analysis

Targeted next-generation sequencing (NGS) using an inherited eye disease panel identified two heterozygous variants in the HPS1 gene (NM_000195.5): c.397G > T, p.(Glu133Ter) in exon 5 and c.972dup, p.(Met325HisfsTer128) in exon 11. Both variants were classified as pathogenic according to the ACMG/AMP guidelines (PVS1, PS3, PM2, PP3). Segregation analysis confirmed the alleles are in trans (biparental inheritance), consistent with the autosomal recessive inheritance pattern of HPS-1.

### 3.3 Differential diagnosis

The diagnostic workup strictly followed the International Society on Thrombosis and Haemostasis (ISTH) diagnostic algorithm for inherited platelet function disorders (IPFD) (8). According to this algorithm, the preliminary laboratory investigation requires the exclusion of thrombocytopenia and von Willebrand disease (VWD). Therefore, immune thrombocytopenia (ITP) and VWD were excluded in the first step based on a normal platelet count, normal routine coagulation tests, normal factor VIII and VWF antigen levels, and the absence of autoimmune markers.

Following this preliminary evaluation, and given the combination of oculocutaneous albinism and normal thromboelastometry profiles, targeted high-throughput genetic sequencing (NGS) was deployed as the primary second-line definitive tool to circumvent the methodological limitations and inaccessibility of platelet transmission electron microscopy.

The subsequent differential diagnosis focused on non-syndromic forms of oculocutaneous albinism (OCA) and other syndromic platelet disorders:

- **Non-syndromic Oculocutaneous Albinism (OCA):** Given the unusual ophthalmological presentation without severe classic visceral HPS symptoms at this age, isolated OCA types (e.g., OCA1, OCA2, OCA4) were strongly considered (10). However, these isolated forms do not feature platelet storage pool deficiency or systemic bleeding diatheses, which prompted further syndromic evaluation.
- **Other HPS subtypes (HPS-2 to HPS-11):** These subtypes are phenotypically similar to HPS-1 and require molecular genetic testing for differentiation (3). Pathogenic variants in HPS2, HPS3, HPS4, and related genes affect the BLOC-1 and BLOC-2 complexes, resulting in variable visceral involvement.
- **Chédiak-Higashi syndrome (LYST):** This condition is characterized by giant lysosomes, progressive immunodeficiency, neurologic impairment, and an accelerated phase. Our patient presented with age-appropriate immunologic and neurologic findings, excluding this diagnosis.
- **Griscelli syndrome (RAB27A, MYO5A, MLPH):** Presents with primary immunodeficiency and neurologic deficits, but lacks visceral fibrosis. This syndrome was ruled out by the genetic confirmation of HPS-1.

## 4 Therapeutic intervention, follow-up and outcomes

Following clinical and molecular confirmation of HPS-1, a multidisciplinary management strategy was initiated. The patient and family received structured education regarding the strict avoidance of NSAIDs, platelet-inhibiting drugs, and trauma-prone contact activities. Standby hemostatic prophylaxis with oral tranexamic acid (20 mg/kg) was prescribed for acute bleeding episodes.

Ophthalmological management centered on individualized refractive correction tailored to congenital nystagmus and foveal hypoplasia, which was well tolerated. No specialized medical or surgical ophthalmological interventions were required. Additionally, the patient was referred for baseline pulmonary and gastrointestinal assessments. Although pulmonary fibrosis and granulomatous colitis are not typically expected at this young age, anticipatory multidisciplinary surveillance is a crucial component of HPS-1 management.

Longitudinal follow-up focused on monitoring best-corrected visual acuity (BCVA), nystagmus stability, and iris transillumination. BCVA improved in the right eye from 0.10 to 0.15, while remaining stable in the left eye at 0.15. Repeat slit-lamp biomicroscopy confirmed persistent absence of iris transillumination (grade 0/5), validating the reproducibility of this phenotypic feature. Horizontal fine-beating nystagmus remained clinically unchanged without progression. Baseline macular optical coherence tomography (OCT) confirmed Grade 3 foveal hypoplasia according to the Thomas et al. classification (11).

Overall clinical outcome was characterized by functional visual stability, absence of severe hemorrhagic crises, and complete tolerance of optical correction without necessitating intervention escalation.

## 5 Discussion

HPS-1 is caused by a deficiency in the BLOC-3 complex, which is essential for intracellular protein trafficking to LROs, including melanosomes and platelet dense granules (3). The lack of functional *δ*-granules leads to a defect in primary hemostasis. Clinically, this manifests as spontaneous ecchymoses and epistaxis despite a normal platelet count and intact coagulation pathways. For women of reproductive age, this commonly results in HMB (12, 13).

The presented case demonstrates an unusual ophthalmological phenotype in molecularly confirmed HPS-1. Although variability in iris pigmentation has been described (5, 14), our patient presented with a complete absence of iris transillumination (score 0/5) alongside marked posterior ocular albinism (albinotic fundus, foveal hypoplasia). Furthermore, HPS-1 generally presents with more pronounced iris transillumination compared to other HPS genotypes (14). Notably, Bobreshova et al. (2024) described another patient harboring the identical compound heterozygous HPS1 variants (c.397G > T/c.972dupC) who exhibited grades 1–2 iris transillumination (5). This direct comparison illustrates that the severity of ophthalmological manifestations is not strictly determined by the HPS1 genotype alone, pointing to the influence of additional genetic, epigenetic, or developmental modifying factors.

The gynecological and renal findings constitute additional unusual features of this case. Bilateral renal pelvic dilatation is likely an incidental, transient pediatric finding, as structural renal anomalies are not classically associated with HPS (3). Similarly, the marked uterine hypoplasia and afollicular ovaries in a 10-year-old suggest a constitutional delay of puberty rather than a direct manifestation of the BLOC-3 deficiency. However, reporting these coincident findings is essential to determine if structural or developmental genitourinary delays represent a broader, yet-unrecognized part of the HPS-1 phenotypic spectrum or merely independent incidentalomas.

The presented case demonstrates a phenotypic dissociation between the degree of pigmentation in the anterior and posterior segments of the eye: preserved iris pigmentation with an absence of its transillumination does not preclude a severe foveal impairment or an albinotic fundus (14). This observation aligns with existing data on the significant clinical and genetic heterogeneity of HPS (1, 3, 5). The practical significance of our findings is that the absence of iris transillumination should not be considered an exclusionary criterion for HPS-1, especially when combined with congenital nystagmus, foveal hypoplasia, albinotic fundus changes, and systemic signs of a bleeding diathesis. In such cases, a comprehensive ophthalmological evaluation and molecular genetic verification of the diagnosis remain essential (1, 5).

The limited availability of platelet transmission electron microscopy (TEM) in the Russian Federation complicates routine diagnostics (5). Since light microscopy cannot reliably quantify dense granules, evaluation requires specialized aggregometry, PFA-100/200 testing, or genetic confirmation. In this case, the identified HPS1 variants match established databases (ClinVar, HGMD), confirming the molecular diagnosis.

### 5.1 Management strategy and prevention of HMB

As the patient is 10 years old with delayed pubertal development (Tanner I) and marked uterine hypoplasia, her future menstrual phenotype cannot yet be reliably predicted. Therefore, current gynecological management focuses strictly on anticipatory counseling rather than the treatment of an established clinical problem. Consistent with ISTH-SSC and ACOG guidance (8, 15), the family was educated on recognizing severe hemorrhage and accessing emergency care. Prophylactic strategies for heavy menstrual bleeding, such as tranexamic acid (25–50 mg/kg/day) or hormonal suppression (12, 13, 15), will be evaluated only upon menarche. In the context of HPS-1, equal emphasis must be placed on anticipatory pulmonary and gastrointestinal surveillance (2).

The limited availability of platelet transmission electron microscopy (TEM) in the Russian Federation complicates routine diagnostics. An emerging and highly practical diagnostic alternative is the immunofluorescence analysis of peripheral blood smears for the assessment of platelet granule proteins (e.g., LAMP-1/CD107a or CD63), as validated by Zaninetti and Greinacher (9). This approach has the distinct advantage of requiring only a small volume of blood on a standard smear, which can easily be shipped over long distances to specialized laboratories. In the absence of TEM or immunofluorescence, molecular genetic confirmation (NGS) remains the definitive diagnostic standard. In this case, the identified *HPS1* variants match established databases (ClinVar, HGMD), confirming the molecular diagnosis.

## 6 Conclusion

This case confirms the clinical and molecular diagnosis of HPS-1 in a 10-year-old female, highlighting an unusual clinical phenotype. The complete absence of iris transillumination, especially in comparison to patients sharing the identical compound heterozygous *HPS1* genotype, broadens the known phenotypic spectrum and underscores the influence of modifying factors. We interpret the observed uterine hypoplasia and delayed ovarian maturation as constitutional, age-related findings requiring independent gynecologic surveillance. Management of HPS-1 must include routine visceral screening, formal hemostatic profiling, and genetic counseling. Ultimately, reporting atypical presentations is vital to improve our understanding of the clinical variability of rare disorders such as HPS. Centralized national registries serve as a valuable tool to aggregate these rare clinical nuances and standardize multidisciplinary diagnostic algorithms.

## 7 Patient perspective

For years, we were very worried about our daughter because she bruised so easily and would bleed for a long time, even though her regular blood tests always looked fine. Finally getting the genetic test and hearing the name Hermansky-Pudlak syndrome changed everything for us. It is scary to find out your child has a rare disease, but it was also a huge relief to finally have answers. Now we know exactly what we are dealing with. We feel much more prepared for her teenage years because we know how to keep her safe and prevent bad bleeding in the future.

## Data Availability

The original clinical contributions presented in the study are included in the article; further inquiries can be directed to the corresponding authors. The identified genetic variants in the HPS1 gene (NM_000195.5) are publicly available in the NCBI ClinVar repository under accession numbers VCV000523626 (https://www.ncbi.nlm.nih.gov/clinvar/variation/523626/ for c.397G>T) and VCV000005279 (https://www.ncbi.nlm.nih.gov/clinvar/variation/5279/ for c.972dup). Raw next-generation sequencing (NGS) data are not publicly available due to patient privacy, ethical restrictions involving a minor, and legal regulations in the Russian Federation, but de-identified data are available from the corresponding authors upon reasonable request.
